# Governing the AI–biotech convergence: The rapid progress in and the dual-use nature of biotechnology and AI requires adaptive and resilient regulatory frameworks to address potential risks

**DOI:** 10.1038/s44319-025-00628-w

**Published:** 2026-01-03

**Authors:** Benjamin D Trump, Christopher L Cummings, Beth Ellinport, Stephanie Galaitsi, Thomas Janisko, Elizaveta Pinigina, Hannah Herzig, Cindy S Groff-Vindman, Markus Schmidt, Gerald Epstein, Ruth Mampuys, Christian Haggenmiller, Tatyana Novossiolova, Travis Tubbs, James H Lambert, Alexander Titus, Igor Linkov

**Affiliations:** 1 Tor Intelligence, LLC, Raleigh, NC USA; 2 U.S. Army Corps of Engineers Engineering Research Development Center, Concord, MA USA; 3 Credere Associates, LLC, Westbrook, ME USA; 4 U.S. Army Corps of Engineers U.S. Public Health Service, Washington, DC, USA; 5 Directorate-General Health Emergency Preparedness and Response Authority (HERA), European Commission, Brussels, Belgium; 6 Army Research Laboratory, Washington, DC, USA; 7 Biofaction, Vienna, Austria; 8 RAND Corporation, Arlington, VA USA; 9 The Netherlands Scientific Council for Government Policy, South Holland, Netherlands; 10 German Institut for Defense and Strategic Studies, Hamburg, Germany; 11 Center for the Study of Democracy, Sofia, Bulgaria; 12 Department of the Air Force/United States Air Force Academy, Colorado Springs, CO USA; 13 University of Virginia, Charlottesville, VA USA; 14 In Vivo Group, Washington, DC, USA

**Keywords:** Biotechnology & Synthetic Biology, Economics, Law & Politics

## Abstract

The convergence of artificial intelligence with biotechnology accelerates innovation but also introduces significant ethical and security risks. These require adaptive, flexible governance strategies that ensure that breakthroughs can be managed responsibly while mitigating potential risks.

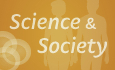

## MAIN

Biotechnology involves the deliberate manipulation of biological systems, organisms and processes to develop novel products in medicine, agriculture, environmental management or chemical engineering. As such, it is a dual-use technology that can be applied both for beneficial goals and misapplied to threaten public health or national security, whether purposefully or inadvertently (NASEM, 2025). Additionally, cultural values often define what are acceptable applications of biotechnology, which makes ethical discussions necessary and challenging. These difficulties are compounded by the fact that technology in general transcends jurisdictional and geographic borders, complicating efforts to enforce uniform and global standards. Moreover, the rapid progress of scientific research and its applications increasingly outpaces governments’ abilities to pass efficient regulations so as to ensure safety and security. For instance, gene editing technology (Fineran and Charpentier, 2012) quickly challenged international frameworks on the regulation of genetically modified organisms.

The convergence of biotechnology and artificial intelligence (AI) stands to accelerate innovation, with potentially substantial benefits but it also introduces a spectrum of new safety and security risks (Moghadasi et al, 2024a, 2024b). By way of example, a recent study using machine learning discovered 161,979 unrecognized viruses from publicly available databases (Hou, et al, 2024). This power necessitates cautious governance to balance innovation with safety and security.

While risks have long accompanied technologies such as gene editing, Design, Build, Test, Learn (DBTL) automation or whole-genome synthesis, DL introduces qualitatively different concerns. DL systems can generate results at a scale and speed that far exceed traditional discovery pipelines, for instance, identifying protein structures or genomic variants within days, which would have taken years to do experimentally. At the same time, the “black box” nature of DL models means that their reasoning may be opaque even to the developers, raising epistemic risks that were less pronounced in earlier technologies. In biomedical research, applications such as antibody design, drug repurposing or vaccine optimization may advance more rapidly but with less transparency, creating opportunities for misuse. This places DL-enabled biotechnology outside the scope of risks that existing governance frameworks were designed to address.

### Deep learning (DL) in biotechnology

DL, a subset of AI, is a particularly powerful tool for biotechnology owing to its ability to learn from large, unstructured biological data, such as genomic sequences, protein structures or medical images (Mienye and Swart, 2024), and uncover subtle patterns and relationships that other AI methods might miss. The integration of DL has significantly accelerated the Design Build, Test, Learn (DBTL) cycle in automation and expedited biological discovery and engineering (Groff-Vindman et al, 2025) as it leverages computational power, a bottleneck in molecular biology. For instance, DL facilitates rapid design through advanced pattern recognition, streamlines the build phase with optimized genetic constructs, automates data analysis during testing, and enhances learning by identifying key insights for continuous improvement. DL’s application in biotechnology is set to transform healthcare, agricultural development and environmental management (Beardall et al, 2022).

The rapid integration of DL in biotechnology presents regulatory challenges as existing frameworks struggle to keep pace with the rate of innovation (Batalis et al, 2024; Groff-Vindman et al, 2025). DL also carries risks beyond those typically associated with biosafety and security. Its “black box” nature introduces epistemic risks where researchers may implement AI-designed systems without fully understanding their functional mechanisms, creating potential for unpredictable properties of engineered organisms (Wheeler et al, 2024). Likewise, DL and AI automation could open risks of malicious manipulation or cybersecurity threats regarding the development and use of medical devices and treatments (O’Brien and Nelson, 2020). Researchers have already demonstrated successful weaponization of AI-driven drug discovery systems by inverting optimization objectives from minimizing to maximizing toxicity, thereby generating 40,000 toxic molecules and a nerve agent within several hours (Urbina et al, 2022).

If left ungoverned, DL could be misapplied in ways that increase biological risk, through the redesign of known toxins or the generation of biomolecules that evade traditional DNA synthesis screening. Systems designed for protein-structure prediction, drug–target identification, or metabolic-pathway optimization could theoretically be inverted or subtly altered to prioritize properties such as toxicity, virulence or immune evasion. For example, algorithms that accelerate antibody discovery could, if misused, also propose immune-evasive viral proteins, while DL models trained on drug–target interactions could be redirected to design molecules that disrupt host immune responses. However, recent assessments from The Age of AI in the Life Sciences report emphasize that these capabilities remain limited (NASEM, 2025). Data scarcity, biological complexity and the need for extensive experimental validation still pose significant constraints. The risks of misuse are not abstract but are presently bounded by technical and data limitations, underscoring the importance of governance before these constraints erode (NASEM, 2025).

### Challenges for governance

The governance of biotechnology and artificial intelligence has long struggled with what Collingridge (1980) termed the “dilemma of control.” At an early stage, the implications of new technologies are uncertain, making it difficult to design appropriate safeguards. At later stages, once a technology is widely adopted and embedded in institutions, markets and social practices, interventions become far more difficult to implement. This problem is acute at the convergence of biotechnology and DL. By accelerating the DBTL cycle and democratizing access to sophisticated design tools, DL shortens the period during which policymakers can deliberate over potential consequences and come up with appropriate preventive measures.

The implications are evident in biotechnology’s recent history. Genome-editing tools such as CRISPR/Cas advanced rapidly from basic discovery to widespread use, leaving regulatory debates and ethical discussions trailing behind. With DL-enabled biotechnology, the pace is even faster. This creates pressure to make early decisions about oversight, but with limited ability to forecast technical feasibility, misuse potential or societal impacts. In this environment, governments face the risk of either over-regulating technologies before their benefits are fully realized or delaying action until risks have already materialized.

Two dominant policy reactions illustrate this tension. At one extreme, protectionist or risk-averse approaches seek to constrain DL-biotechnology at the outset. Regulators may restrict access to datasets, algorithms or laboratory materials on the grounds that they could be misapplied for harmful purposes (Herdegen, 2018). However, overly strict regulation of DL-based design tools could inhibit the development of new therapeutics, biomaterials or climate-change resilient crops. Moreover, restrictive policies are increasingly impractical as the tools of biotechnology become more distributed and affordable. Attempting to regulate computational methods or restrict access to open-source code may encourage noncompliance, fragment global research collaboration, and foster “safe havens” where development proceeds with fewer safeguards.

At the other extreme, laissez-faire or risk-tolerant approaches delay regulatory intervention to avoid hindering innovation. This permissive stance can foster rapid progress, but it also increases the risk of unintended consequences. DL-biotechnology tools applied without adequate oversight may accelerate the release of poorly characterized organisms into the environment, amplify risks of off-target genetic effects or enable actors with malicious intent to pursue sophisticated bioweapons (O’Brien and Nelson, 2020). By postponing governance, states risk losing the ability to shape norms and safeguards, while public trust may erode if negative outcomes occur.

Both extremes highlight the inadequacy of traditional governance approaches when confronted with rapidly evolving dual-use technologies. The challenge lies in designing frameworks that allow beneficial applications to flourish while constraining misuse in ways that are flexible enough to adapt as new knowledge emerges. This requires moving beyond binary choices of early restriction or delayed response, and instead adopting iterative, adaptive strategies that can evolve alongside the technology.

Complicating this picture are the uneven global dynamics and regulations of biotechnology. Nations differ in their regulatory capacities, levels of investment and cultural attitudes toward risk. Differential regulation can fragment global research efforts, as scientists navigate incompatible standards or seek permissive jurisdictions for sensitive work. Efforts to harmonize governance across borders are therefore essential, though politically challenging. Without some degree of coordination, the benefits of DL-biotechnology may concentrate unevenly, while risks diffuse globally. Addressing these challenges requires flexible governance strategies that neither freeze innovation nor abandon precaution, but instead recognize the need for adaptive, anticipatory oversight that can evolve as the technologies themselves evolve.

### Adaptive governance frameworks

Addressing the governance paradox, where it is too difficult to foresee the full implications of these technologies, yet urgent to establish control, requires moving away from rigid or reactive approaches. Traditional mechanisms of control, whether overly restrictive or permissive, have proven insufficient for technologies that evolve at the pace of biotechnology. What is needed are governance frameworks that are adaptive, anticipatory and resilient, able to adapt to innovation and to respond to new risks as they arise.

Adaptive measures differ from traditional rules or regulations because they are explicitly designed to evolve in step with scientific progress. Instead of setting fixed regulations that may become obsolete as technologies advance, adaptive measures rely on iterative updates, stakeholder engagement and feedback from ongoing practice. For biomedical scientists, this resembles updating experimental protocols in response to new findings: governance itself becomes an experiment that learns over time. This concept is novel in the biotechnology domain, where regulation has historically emphasized stability and legal certainty.

Debates about DL-biotechnology are shaped as much by values and expectations as by technical realities. Scholars and policymakers frequently interpret convergence through different outlooks of optimism and pessimism (Torgersen and Schmidt 2013). Some take a nonspecific optimism, assuming that rapid innovation will generate widespread benefits in health, agriculture and environmental sustainability. Others argue from a position of specific optimism, suggesting that expertise and deliberate stewardship can ensure that new technologies will deliver breakthroughs while also protecting against deliberate misuse. In contrast, nonspecific pessimists emphasize uncertainty, contending that inadequate knowledge will inevitably lead to harmful outcomes. Specific pessimists warn that convergence will be exploited by malicious actors, pointing to risks such as engineered pathogens or breaches of privacy (O’Brien and Nelson, 2020).

Despite these divergent framings, all share a recognition that uncertainty is unavoidable. Moderates within this debate emphasize a “troubleshooter” approach: advocating for iterative oversight, technical fixes and governance that remains flexible without ignoring risks or constraining beneficial applications (Undheim, 2024). This framing is valuable because it provides a pathway for practical action in an environment of uncertainty.

Adaptive governance typically combines multiple instruments, described as a mix of “hard law” and “soft law” (Shaffer and Pollack, 2008). Hard-law mechanisms, including national legislation and international treaties such as the Biological Weapons Convention (BWC) or domestic regulatory frameworks like FDA biotechnology oversight, provide binding authority with clear compliance requirements and formal sanctions, but are often slow to negotiate and difficult to amend (Trump, 2017). They are appropriate for addressing well-characterized risks, such as biological weapons development, but they are poorly suited to technologies that evolve rapidly (Batalis et al, 2024).

Soft-law instruments, including voluntary guidelines, standards and professional codes of conduct, such as the International Gene Synthesis Consortium’s DNA screening protocols, can provide flexibility and stakeholder engagement, involving scientists, industry representatives, civil society and governments (Trump et al, 2020). These guidelines can also be revised more easily and often emerge from multi-stakeholder collaborative processes; however, they lack formal binding mechanisms and comprehensive coverage across all relevant actors (Trump et al, 2020).

Soft-law mechanisms and adaptive governance frameworks demonstrate greater efficacy in managing biotechnology risks than traditional hard-law approaches, particularly when addressing novel challenges of rapidly emerging technological innovations (Wheeler et al, 2024). The temporal mismatch between technological advancement and regulatory response creates what Trump et al, (2023) characterize as an “innovation dilemma,” wherein traditional hard-law frameworks prove structurally inadequate for managing risks that evolve faster than legislative processes can accommodate.

Empirical evidence from successful soft-law implementations demonstrates that voluntary industry standards can achieve broader sectoral coverage and prove more rapidly implementable than mandatory regulatory frameworks while fostering greater stakeholder engagement (Trump et al, 2020; Pannu et al, 2025). Contemporary examples include the Gene Synthesis Consortium’s implementation of machine learning algorithms to detect potentially hazardous sequences that may evade traditional homology-based screening, and the emergence of “AI red-teaming” initiatives wherein security researchers deliberately attempt to misuse AI design tools to identify vulnerabilities before malicious actors exploit them (Urbina et al, 2022). More recently, another approach emerged that recognizes the end user within the defense strategy. This approach is known as Violet Teaming, integrating traditional security models in real user contexts (Titus and Russell 2023). Examining how these DL models interact with human behavior is similar to the Partnership on AI’s biosafety working groups’ development of safety standards for biological applications of AI, incorporating continuous stakeholder consultation, regular protocol updates aligned with emerging research findings and flexible implementation guidelines that accommodate diverse institutional contexts (Pannu et al, 2025).

### National versus international regulatory frameworks

The jurisdictional complexities inherent in regulating DL-enabled biotechnologies create fundamental tensions between national imperatives and the necessity for coordinated international oversight. Both hard and soft-law frameworks present distinct challenges in appropriately governing AI-enabled biotechnology across borders. Hard-law mechanisms encounter significant obstacles due to their inherent inflexibility and the requirement for formal international consensus. Consequently, updating existing hard-law frameworks such as the BWC presents substantial procedural challenges, including the requirement for consensus among all 183 state parties, a timeline incompatible with the rapid pace of technological evolution in biotechnology (Trump et al., 2021).

At the national level, regulation of specific applications, exemplified by DNA synthesis order screening requirements, implemented in countries such as the USA, Germany or Singapore, creates legally binding obligations with clear compliance frameworks and penalty structures that voluntary industry standards cannot replicate, while maintaining sufficient adaptability (Oye 2012).

Conversely, soft-law frameworks can prove effective at the international scale for DL-enabled biotechnologies due to their inherent flexibility and typical rapidity of development and implementation. However, they suffer from inadequate enforcement mechanisms and limited democratic legitimacy, as these governance approaches may lack the transparency, accountability and public participation processes that characterize formal legislative frameworks (Trump et al, 2023). Given the fundamental uncertainty surrounding DL-biotechnology, the respective trade-offs of each regulatory approach must be carefully balanced to create governance frameworks that maintain both effectiveness and adaptive capacity.

The BWC exemplifies hard law: it is binding, global in scope and explicitly prohibits the development of bioweapons; yet its treaty structure makes adaptation to emerging risks slow and politically complex. DNA synthesis screening, implemented through both national regulations and industry standards such as the International Gene Synthesis Consortium protocols, functions as a more adaptive measure. Screening requirements can be rapidly revised as new threat sequences are identified, and companies can adopt improved detection algorithms, including DL-based methods. This contrast illustrates how hard and soft-law mechanisms operate at different scales and speeds, and why both are necessary to govern DL in biotechnology effectively.

### Overcoming the “too soon to know, too late to act” policy paradox

Concerns arising from DL-biotechnology can be grouped into three categories: security, safety and efficacy. Security addresses the potential for deliberate misuse, such as the creation of bioweapons. Safety concerns reflect the risk of unintended harms, such as ecological disruption or off-target genetic effects from poorly characterized interventions. Efficacy focuses on whether technologies perform as intended, recognizing that technical limitations or unforeseen interactions may compromise the expected results (Pei et al, 2022). Categorizing risks in this manner allows for more proportional oversight and prevents the conflation of highly specific threats with general anxieties. Hazard, exposure and effects assessments that are common in environmental and health domains can be applied to DL-biotechnology, strengthening foresight and improving decision-making.

Traditional risk management emphasizes prevention and defense: reducing the probability of adverse events and mitigating impacts once they occur. DL-biotechnology requires equal attention to detection and delay. Detection involves surveillance and horizon scanning systems that can identify anomalies, suspicious activity or unintended outcomes. DL itself can be used to monitor scientific publications, patent filings or social media to detect signals of potential misuse or risky applications (Wang et al, 2021a, 2021b). Delay refers to mechanisms such as phased rollouts, controlled environments or temporary moratoriums that provide additional time to evaluate risks before widespread deployment.

A further principle is human-in-the-loop oversight. DL models often operate as “black boxes,” producing results that may be difficult for experts to interpret. Without meaningful human engagement, it becomes difficult to ensure accountability, legitimacy or ethical alignment. Embedding human judgment into automated design, testing, and risk-assessment processes allows ethical considerations and societal values to remain central. This approach also creates opportunities for participatory governance, involving diverse stakeholders in the evaluation of trade-offs. Transparency and legitimacy can be improved when experts, policymakers and the public are all engaged in shaping outcomes.

Global coordination is another priority, but comprehensive global agreements are difficult given geopolitical fragmentation. A more pragmatic pathway involves international networks and coalitions of states that agree on shared standards, data exchange, and best practices. These coalitions can serve as clearinghouses for new risks, platforms for governance innovation and forums for harmonization. They need not include all countries immediately: smaller groups of committed actors can establish norms and practices that others may later adopt (Holland et al, 2024).

Adaptive governance must also embed continuous learning and feedback. Governance arrangements should be evaluated regularly for effectiveness and unintended outcomes, with mechanisms for revision when new evidence emerges. Governance should be modular, allowing individual measures to be updated or replaced without overhauling the entire system. DL itself can play a role in this process by modeling potential regulatory outcomes or by analyzing large datasets to identify emerging patterns of risk (Moghadasi et al, 2024a; Moghadasi et al, 2024b).

Taken together, these elements form the foundations of adaptive governance for DL-biotechnology: multi-stakeholder participation, the integration of hard and soft law, categorization of risk, resilience strategies, global coordination and mechanisms for continuous learning. Such frameworks do not resolve the Collingridge dilemma or the “too soon, too late” paradox (Collingridge, 1980). They do, however, provide ways to mitigate its effects by embedding flexibility into governance systems to capture the benefits of DL-biotechnology while reducing the likelihood and severity of harmful consequences.

### Building opportunities for resilience

Governments traditionally rely on four strategies to address risk: prevention, detection, delay, and defense. Prevention seeks to reduce the likelihood of adverse events before they occur, for example, by establishing biosafety protocols, security measures and review standards. Detection emphasizes surveillance to identify early signs of misuse or unintended consequences, drawing on both traditional monitoring and DL-enabled analytical tools (Wang et al, 2021a, 2021b). Delay strategies intentionally slow the deployment of novel applications, providing time for assessment through moratoriums, phased approvals or containment measures. Defense focuses on responding once harms have occurred, including countermeasures such as vaccines, antidotes or responses by public health agencies.

These strategies provide a useful foundation for governing the convergence of DL and biotechnology, but they must be interpreted through a resilience lens. Prevention and defense reflect a risk-based approach to avoid or contain hazards. Detection and delay introduce resilience-based thinking, emphasizing adaptability and recovery when complete control is not possible. For DL-biotechnology, where opacity and speed make precise risk prediction difficult, resilience strategies are particularly important. By strengthening detection and delay, governments create the capacity to adjust as technologies mature rather than locking themselves into premature approvals or overly rigid prohibitions.

Hard and soft law both play roles in implementing resilience strategies. Defense, for example, often requires hard-law frameworks that establish liability, regulate access to sensitive materials and criminalize misuse. Detection and delay may rely on combinations of measures, such as surveillance mandates or reporting requirements, and softer instruments like voluntary standards or guidelines for responsible research conduct. Soft-law approaches are especially well-suited for fast-moving areas because they can be updated regularly without imposing inflexible rules (Shaffer and Pollack, 2008).

Resilience-based strategies also highlight the importance of international coordination. DL-biotechnology research and applications circulate across borders, and the consequences of accidents or malicious use are not confined to a single jurisdiction. Yet global consensus on governance is unlikely given political and economic fragmentation. A more feasible path is to build networks of committed states that share information, develop common monitoring practices and coordinate risk categorization (Holland et al, 2024). Over time, they can provide templates for wider adoption and eventual harmonization, even without a universal treaty.

Resilience depends on participatory engagement. Public trust is easily undermined if governance is perceived as either obstructing beneficial innovation or failing to prevent harmful outcomes. Inclusive consultation with scientific communities, industry and civil society can improve legitimacy and foster alignment of governance with societal values (Torgersen and Schmidt 2013). Approaches such as Violet Teaming operationalize this human-centered perspective by examining how systems interact with human behavior, identifying gaps where policy or design can fail. Integrating end-user contexts into security protocols bridges the gap between technical defenses and the practical realities of how DL-biotechnology tools are used. Furthermore, proactive dialog about the benefits and risks of DL-biotechnology can reduce polarization and build shared understanding across diverse perspectives. Participatory mechanisms also improve foresight by surfacing concerns and knowledge that may not be visible to technical experts alone.

DL-biotechnology convergence cannot be governed by relying solely on past models of oversight. The pace of innovation requires adaptive frameworks that can evolve alongside science, anticipating risks without foreclosing opportunities. The most effective path is not a choice between optimism or pessimism, nor between laissez-faire permissiveness and precautionary prohibition, but a balance that allows innovation to proceed while maintaining safeguards against misuse and unintended consequences. By investing in resilience-based strategies, strengthening coordination and embedding continuous learning, policymakers and practitioners can help ensure that this convergence advances human welfare without undermining security or ethics. Adaptive governance will not resolve all uncertainties, but it can provide a dynamic foundation for managing them in a responsible and constructive way (Trump et al, 2020).

## Supplementary information

Peer Review File
