# Supplementary material for: Governing the AI–biotech convergence: The rapid progress in and the dual-use nature of biotechnology and AI requires adaptive and resilient regulatory frameworks to address potential risks
Source: EMBO Rep. 2026 Jan 3;27(2):259–64. doi: 10.1038/s44319-025-00628-w (PMC12852783; doi:10.1038/s44319-025-00628-w)
Supplement: Supplementary file 1 — Peer Review File [file 44319_2025_628_MOESM1_ESM.pdf]

EMBO reports

Governing the AI-Biotech Convergence: Adaptive Strategies for Uncertain Landscapes

Benjamin D Trump, Christopher L. Cummings, Beth Ellinport, Stephanie Galaitsi, Thomas Janisko, Elizaveta Pinigina, Hannah Herzig, Cindy S. Groff-Vindman, Markus Schmidt, Gerald Epstein, Ruth Mampuys, Christian Haggenmiller, Tatyana Novossiolova, Travis Tubbs, James H. Lambert, Alexander Titus, and Igor Linkov

Corresponding author(s): Beth Ellinport (Beth.d.ellinport@usace.army.mil)

Review Timeline:

Submission Date:	9th Sep 25
Editorial Decision:	15th Sep 25
Revision Received:	24th Sep 25
Accepted:	24th Oct 25

Editor: Holger Breithaupt

Transaction Report: This article was editorially reviewed.
